# ssc-miR-185 targets cell division cycle 42 and promotes the proliferation of intestinal porcine epithelial cell

**DOI:** 10.5713/ajas.20.0325

**Published:** 2020-10-12

**Authors:** Wei Wang, Pengfei Wang, Kaihui Xie, Ruirui Luo, Xiaoli Gao, Zunqiang Yan, Xiaoyu Huang, Qiaoli Yang, Shuangbao Gun

**Affiliations:** 1 College of Animal Science and Technology, Gansu Agricultural University, Lanzhou, Gansu 730070, China; 2 Gansu Research Center for Swine Production Engineering and Technology, Lanzhou, Gansu 730070, China

**Keywords:** ssc-miR-185, Cell Division Cycle 42 (CDC42), Target Relationship, Proliferation, Intestinal Porcine Epithelial Cell (IPEC-J2)

## Abstract

**Objective:**

microRNAs (miRNAs) can play a role in a variety of physiological and pathological processes, and their role is achieved by regulating the expression of target genes. Our previous high-throughput sequencing found that ssc-miR-185 plays an important regulatory role in piglet diarrhea, but its specific target genes and functions in intestinal porcine epithelial cell (IPEC-J2) are still unclear. We intended to verify the target relationship between porcine miR-185 and cell division cycle 42 (CDC42) gene in IPEC-J2 and to explore the effect of miR-185 on the proliferation of IPEC-J2 cells.

**Methods:**

The TargetScan, miRDB, and miRanda software were used to predict the target genes of porcine miR-185, and CDC42 was selected as a candidate target gene. The CDC42-3′ UTR-wild type (WT) and CDC42-3′UTR-mutant type (MUT) segments were successfully cloned into pmirGLO luciferase vector, and the luciferase activity was detected after co-transfection with miR-185 mimics and pmirGLO-CDC42-3′UTR. The expression level of CDC42 was analyzed using quantitative polymerase chain reaction and Western blot. The proliferation of IPEC-J2 was detected using cell counting kit-8 (CCK-8), methylthiazolyldiphenyl-tetrazolium bromide (MTT), and 5-ethynyl-2′-deoxyuridine (EdU) assays.

**Results:**

Double enzyme digestion and sequencing confirmed that CDC42-3′UTR-WT and CDC42-3′UTR-MUT were successfully cloned into pmirGLO luciferase reporter vector, and the luciferase activity was significantly reduced after co-transfection with miR-185 mimics and CDC42-3′UTR-WT. Further we found that the mRNA and protein expression level of CDC42 were down-regulated after transfection with miR-185 mimics, while the opposite trend was observed after transfection with miR-185 inhibitor (p<0.01). In addition, the CCK-8, MTT, and EdU results demonstrated that miR-185 promotes IPEC-J2 cells proliferation by targeting CDC42.

**Conclusion:**

These findings indicate that porcine miR-185 can directly target CDC42 and promote the proliferation of IPEC-J2 cells. However, the detailed regulatory mechanism of miR-185/CDC42 axis in piglets’ resistance to diarrhea is yet to be elucidated in further investigation.

## INTRODUCTION

Diarrhea is the main cause of death of newborn and suckling piglets, which brings enormous economic loss to the pig industry [1]. The occurrence of piglet diarrhea is related to a combination of genetic and improper management factors, especially the infection of pathogenic microorganisms, such as *Escherichia coli* [2], *Salmonella* [3], *Clostridium perfringens* [4–6], porcine epidemic diarrhea virus [7], etc. Therefore, it is necessary to search for molecular markers of diarrhea resistance and carry out porcine disease-resistant breeding. MicroRNAs (miRNAs), a class of small and endogenous non-coding RNA molecules with 19–25 nucleotides, can play a key role in post-transcription by binding to the 3′-untranslated region (3′UTR) of target mRNA [8,9]. It can play roles in multiple biological processes, such as the cell proliferation [10], apoptosis [11], tumorigenesis [12], and immune inflammation [13] by suppressing the expression of its target genes.

In our previous study, we researched the expression profiles of the ileum miRNAs of 7 days piglets infected with *Clostridium perfringens* type C using small RNA-Seq, and found that ssc-miR-185 was differentially expressed between the resistant group and susceptible group of diarrhea piglets [14]. It is reported that miR-185 can play important roles in a variety of cancers, covering pancreatic cancer [15], bladder cancer [16], non-small cell lung cancer [17], prostate carcinoma [18], gastric cancer [19], breast cancer [20], hepatocellular carcinoma [21], and colorectal cancer [22]. In addition, miR-185 can also play a regulatory role in response the immune inflammatory. Liu et al [23] analyzed microRNAs in alcoholic liver diseases using microarrays and found that miR-185 can participate in immune response, inflammatory response and glutathione metabolism. Ma et al [24] found that the CCAT1/miR-185-3p/MLCK signaling pathway damages intestinal barrier function and promotes the deterioration of inflammatory bowel disease. Based on this, we speculate that miR-185 also plays an important role in the resistance of piglets to diarrhea infection.

Cell division cycle 42 (CDC42) is one of the members of Rho GTPase family [25]. It is reported that CDC42 regulates cell cytoskeleton and adhesion, cell functions, which are crucial in the development of various cancer diseases [26]. The research reported that miR-137 may directly target CDC42, inducing G1 cell cycle arrest and inhibiting the proliferation and invasion activities of colorectal cancer cells [27]. Moreover, miR-185 is a negative regulator of RhoA and CDC42, and could inhibit the proliferation and invasion of human colorectal cancer cells [28]. However, the function of miR-185/CDC42 in intestinal porcine epithelial cell (IPEC-J2) remains to be determined.

In our current study, the relationship between porcine miR-185 and CDC42 was investigated in IPEC-J2. We predicted the target relationship between miR-185 and CDC42 using bioinformatics software. The mRNA and protein expression level of CDC42 in IPEC-J2 were detected after transfection with miR-185. The luciferase activity of recombinant plasmids was also detected. In addition, the effects of overexpression miR-185 or knockdown CDC42 on proliferation activity of IPEC-J2 were explored. In this study, our results show that porcine miR-185 can directly target CDC42 and promote the proliferation of IPEC-J2 cells.

## MATERIALS AND METHODS

### Ethics statement

All animal experiments were conducted according to the Regulations and Guidelines for Experimental Animals established by the Ministry of Science and Technology (Beijing, China, revised in 2004) and approved by the Committee for Animal Ethics of the College of Animal Science and Technology, Gansu Agricultural University (approval number 2006-398).

### Sample collection and cell culture

The liver tissue samples were collected from three male landrace at six months and stored at −80°C until RNA extraction and as a template for *CDC42* gene 3′UTR amplification. The 293T cells and IPEC-J2 were purchased from BeNa Culture Collection (BNCC, Beijing, China). The cells were cultured in DMEM/F12 medium (HyClone, New York, NY, USA) supplemented with 10% fetal bovine serum (Gibco, Thermo Fisher Scientific, Inc., New York, USA), and 1% penicillin-streptomycin at 37°C and 5% CO_2_. When cell confluence reached 70% to 80%, the transfection is carried out.

### Total RNA extraction and cDNA synthesis

Total RNA was extracted from porcine liver tissues and IPEC-J2 cells using *TransZol* Up reagent (TransGen Biotech, Beijing, China) according to the manufacturer’s instructions. Subsequently, the cDNA was synthesized by reverse transcription using PrimeScript RT reagent kit with gDNA Eraser (TaKaRa, Dalian, China) and stored at −20°C.

### Bioinformatic analysis

Since the miR-185 is highly conserved among different species, the miRNA databases: TargetScan [29] (http://www.targetscan.org/vert_72/), miRDB [30] (http://www.mirdb.org/), and miRanda [31] (http://www.microrna.org/microrna/home.do) online software were used to predict the target genes for porcine miR-185. Based on predictive criteria, being bound to targeted sequences with low free energy of binding and having good complementarity with targeted sequences, CDC42 was selected as a candidate mRNA for follow-up studies.

### Plasmid construction and dual-luciferase reporter assay

To verify the targeting relationship between miR-185 and CDC42, a partial segment of the CDC42 mRNA 3′-UTR (WT) containing the miR-185 binding-sequence was polymerase chain reaction (PCR) amplified using specific primers (Table 1). A mutated segment of the CDC42 mRNA 3′-UTR (MUT) in which the miR-185 binding sequence TCTCTCC was converted to AGAGAGG was obtained using gene synthesis and subcloning (GENEWIZ, Suzhou, China). The PCR products were cloned into the pmirGLO (7,350 bp) dual luciferase reporter vector (Promega, Madison, WI, USA). The recombinant plasmids were confirmed by double enzyme digestion with *Xho* I and *Sal* I (TaKaRa, China) and sequencing.

For transfection, the 293T cells reached 70% to 80% confluences, cells were incubated in 24-well plates. The recombinant plasmids were co-transfected with miR-185 mimics (50 nM) and inhibitor (100 nM) using Lipofectamine 2000 reagent (Invitrogen, Carlsbad, CA, USA) according to the manufacturer’s protocol, respectively. The miR-185 mimics and inhibitor were designed and synthesized by RiboBio Biotech Co., Ltd. (RiboBio, Guangzhou, China). After 48 h post-transfection, the luciferase activity was detected using the Dual Luciferase Reporter Assay System (Promega, USA). In this experiment, the pmirGLO vector was considered as a blank control, mimics NC and inhibitor NC were considered as a negative control. All reactions were performed in triplicate.

### Quantitative polymerase chain reaction

The IPEC-J2 cells were collected after transfection with miR-185 mimics and inhibitor. The quantitative polymerase chain reaction (qPCR) reaction was analyzed using TB Green Premix Ex Taq II (Tli RNaseH Plus) quantitative kit (TaKaRa, China) in Roche LightCycler 480 II instrument (Roche, Penzberg, Germany). The primer sequences are shown in Table 1. The thermal cycle for PCR was performed at 95°C for 30 seconds, 40 cycles at 95°C for 5 seconds and 60°C for 30 seconds. The relative mRNA expression of *CDC42* gene was normalized with β-actin (*ACTB*) gene, and the results were calculated using the 2^−ΔΔCt^ method [32].

### Western blotting

After cell transfection 48 h, total proteins were collected from the treated cells by RIPA buffer (Solarbio, Beijing, China) and quantified using the BCA protein assay kit (Solarbio, Beijing, China). The each group of denatured proteins were loaded into 10% sodium dodecyl sulfate-polyacrylamide gelelectrophoresis, and transferred onto polyvinylidene fluoride membrane. Then, the membranes were blocked in Tris-buffered saline with Tween-20 and incubated with 5% skim milk at room temperature for 1 h. Next the membranes were incubated with primary antibodies (anti-CDC42, bs-3555R, 1:1,000; anti-β-actin, bsm-33036M, 1:1,500, Bioss, Beijing, China) at 4°C overnight. The membranes were then incubated with secondary antibodies (HRP, goat anti-rabbit IgG, bs-0295G-HRP, 1:2,000, Bioss, Beijing, China) for 2 h at room temperature. The final protein bands were visualized by enhanced chemiluminescence, and the gray level of the protein bands was analyzed using ImageJ software (National Institutes of Health, Bethesda, MD, USA).

### Interference RNA synthesis and overexpression vector construction

The interference RNAs used in this experiment were designed and synthesized by GenePharma Company (Shanghai, China). The si-NC was regarded as a negative control. The interference sequences were shown in Table 2. The *CDC42* gene was cloned into pcDNA3.1 (+) vector with *Nhe* I and *BmaH* I restriction sites. The pcDNA-CDC42 overexpression vector was constructed by GENEWIZ Company (Suzhou, China).

### Cell counting kit-8 assay

Cell counting kit-8 (CCK-8, Beyotime, Shanghai, China) was used to detect cell viability. The 2×10^3^ cells per well were inoculated in the 96-well plates and maintained in the incubator for 24 h. Subsequently, the cells were treated with miR-185 mimics, mimics NC, miR-185 inhibitor and inhibitor NC for 24 h, respectively. Add 10 μL of CCK-8 reagent to each well according to the manufacturer’s instructions and incubated for another 4 h. Finally, cell viability was determined by measuring the optical density (OD) at 450 nm.

### Methylthiazolyldiphenyl-tetrazolium bromide assay

The methylthiazolyldiphenyl-tetrazolium bromide (MTT, Beyotime, Shanghai, China) was also used to examine cell viability. The 5×10^3^ cells per well were cultured for 24 h in 96-well plates before treatment with miR-185 mimics, mimics NC, miR-185 inhibitor and inhibitor NC. Then they were incubated for 24 h at 37°C containing 5% CO_2_. Followed by 10 μL of MTT reagent (5 mg/mL) added to per well for another 4 h. The medium was discarded after 4 h of treatment and the formazan crystals were dissolved using 110 μL of dimethyl sulfoxide. The wavelength at 490 nm was selected, and the OD was determined using SkanIt microplate reader (Thermo Fisher Scientific Inc., USA).

### 5-ethynyl-2′-deoxyuridine assay

The BeyoClick EdU Cell Proliferation Kit with Alexa Fluor 555 (EdU, Beyotime, Shanghai, China) was used to detect cell proliferation. After seeding in 24-well plates (5×10^3^ cells per well) for 24 h, the IPEC-J2 cells were transfected. After transfected 24 h, the cells were incubated with 10 μM EdU solution in growth medium for 2 h. Then, the cells stained with Azide 555 solution (red) and Hoechst 33342 (blue). Finally, the results were observed under a fluorescence microscope (Olympus IX71, Tokyo, Japan) with 200× magnification. The EdU positive cells were analyzed with the ImageJ software.

### Statistical analysis

The IBM SPSS Statistics software (version 21.0; IBM, Armonk, NY, USA) was used to analyze the data, all experiments were repeated at least three times. A Student’s t-test was applied to compare two groups and one-way analysis of variance (ANOVA) was performed for multiple groups. All values in this study were expressed as the mean±standard deviation, a p value of less than 0.05 was indicated statistical significance.

## RESULTS

### Predicting targeted mRNA

**Figure 1:**
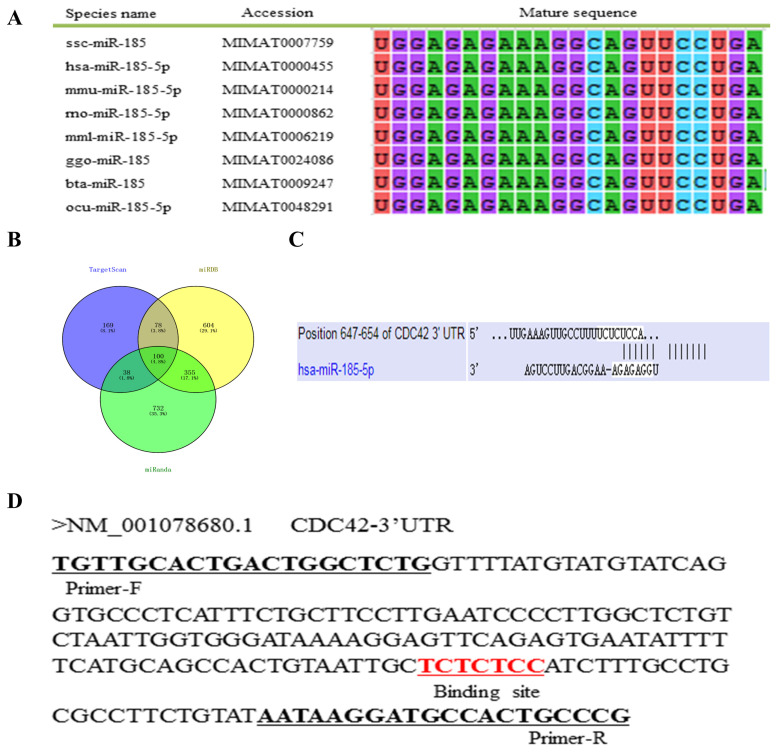
Bioinformatics analysis. (A) miR-185 mature sequences alignment in different species. (B) Venn diagram of target gene intersection. (C) Predicting the binding sites between miR-185 and CDC42 using TargetScan software. (D) *CDC42* gene 3′UTR partial sequences. The black underline indicates the amplification primer and the red underline indicates the binding sites. CDC42, cell division cycle 42; UTR, untranslated region.

To explore the potential mechanism of ssc-miR-185, we performed a multi-sequence alignment analysis of the mature miR-185 sequences in different species and found that the mature sequences of miR-185 was highly conserved in vertebrates (Figure 1A). The targeting mRNAs of miR-185 were predicted using TargetScan, miRDB and miRanda software, and 385, 1,137, and 1,225 target genes were obtained respectively, and 100 common target genes were obtained by the intersection (Figure 1B). It was found that *CDC42* gene 3′UTR can complement and bind to the seed region of miR-185 (Figure 1C). The *CDC42* gene 3′UTR partial sequences contain miR-185 binding sites as showed in Figure 1D.

### Recombinant plasmids identification and luciferase activity detection

**Figure 2:**
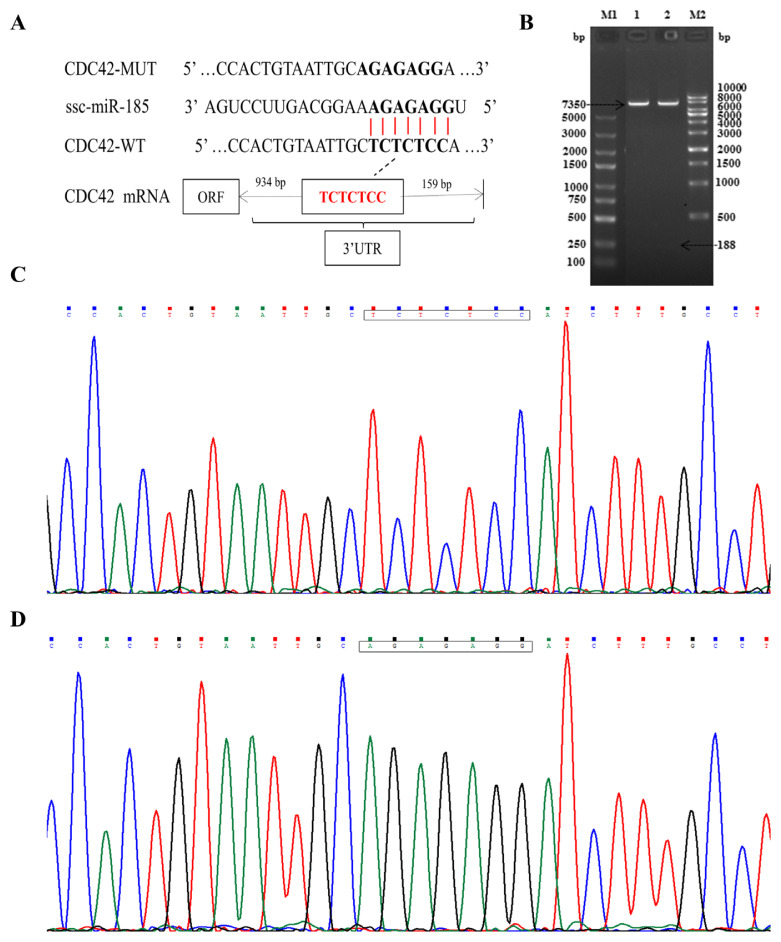
Construction and identification of recombinant plasmids. (A) The location information between miR-185 and CDC42 wild-type and mutant-type. (B) Recombinant plasmid enzyme digestion identification. M1, 5 kb DNA marker; 1 and 2, wild-type and mutant-type recombinant plasmids; M2, 10 kb DNA marker. (C) CDC42-3′UTR-wild type sequencing. The box represents the binding sites. (D) CDC42-3′UTR-mutant type sequencing. The box represents the mutant sites. CDC42, cell division cycle 42; UTR, untranslated region.

**Figure 3:**
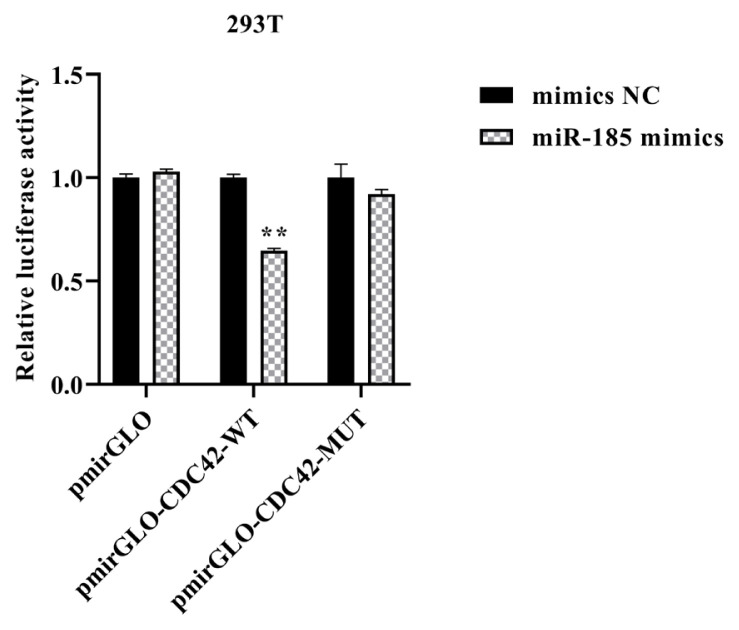
The relative luciferase activity of CDC42-3′UTR. ** Means p<0.01. CDC42, cell division cycle 42; UTR, untranslated region.

Double enzyme digestion and sequencing confirmed that CDC42-3′UTR-WT and CDC42-3′UTR-MUT were successfully cloned into pmirGLO luciferase reporter vector (Figure 2A–2D). The TCTCTCC sequences were successfully mutated to AGAGAGG, without changes to other bases. In order to confirm the role of miR-185 in regulating CDC42-3′UTR, the luciferase activity was detected using the Dual Luciferase Reporter Assay System according to specification. We found that miR-185 mimics remarkably reduced the luciferase activity of the pmirGLO-CDC42-WT (p<0.01), but not that of the pmirGLO and pmirGLO-CDC42-MUT (p> 0.05) (Figure 3).

### Effects of miR-185 on CDC42 expression level in IPEC-J2

**Figure 4:**
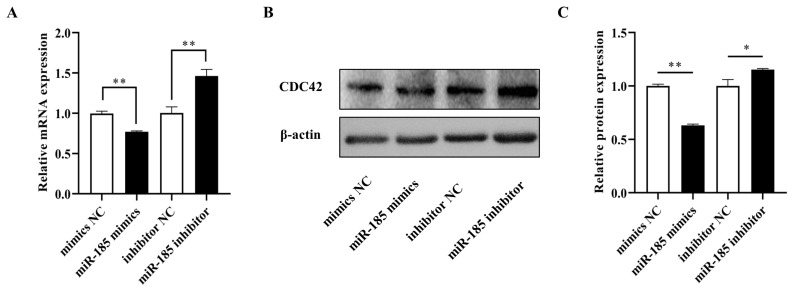
The relative expression level of CDC42. (A) mRNA expression level of CDC42. (B and C) protein expression level of CDC42. CDC42, cell division cycle 42. All ** means p<0.01, * means p<0.05, β-actin was detected as an internal control, n = 3.

To further confirm the effects of miR-185 on CDC42, qPCR and Western blot analyses were used to examine the mRNA and protein expression in IPEC-J2 cells after transfected with miR-185 mimics, mimics NC, miR-185 inhibitor and inhibitor NC, respectively. The results showed that the mRNA and protein expression level of CDC42 was dramatically decreased when transfected with miR-185 mimics than in transfected with mimics NC (p<0.01), however, the CDC42 expression level both mRNA and protein were significantly increased when transfected with miR-185 inhibitor than in transfected with inhibitor NC (p<0.01) (Figure 4A–4C). These results demonstrate that miR-185 directly regulates CDC42 expression.

### miR-185 promotes the IPEC-J2 cell proliferation

**Figure 5:**
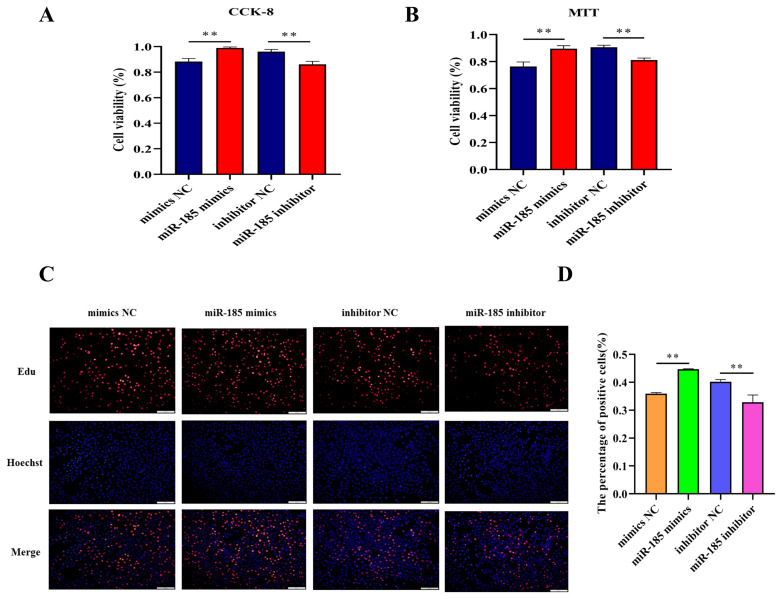
The effect of miR-185 on IPEC-J2 cells proliferation. (A) The results of CCK-8 assay. (B) The results of MTT assay. (C) The results of EdU assay. (D) The count of EdU positive cells. CCK-8, cell counting kit-8; MTT, methylthiazolyldiphenyl-tetrazolium bromide. All ** indicates p<0.01.

In order to explore the effect of miR-185 on IPEC-J2 cell proliferation, CCK-8 assay and MTT assay were used to detect the cell viability after transfected with miR-185 mimics, mimics NC, miR-185 inhibitor and inhibitor NC. We found that overexpression miR-185 enhanced cell viability, while knockdown miR-185 can inhibit cell viability of IPEC-J2 cells. The CCK-8 and MTT assays showed similar expression trends (Figure 5A, 5B). The EdU assay was used to detected cell proliferation, and the results showed that the EdU positive cells were significantly increased after transfected with miR-185 mimics. On the contrary, after transfected with miR-185 inhibitor, the EdU positive cells were significantly reduced. Therefore, we conclude that miR-185 can promote IPEC-J2 cells proliferation.

### Knockdown CDC42 promotes the proliferation of IPEC-J2 cells

**Figure 6:**
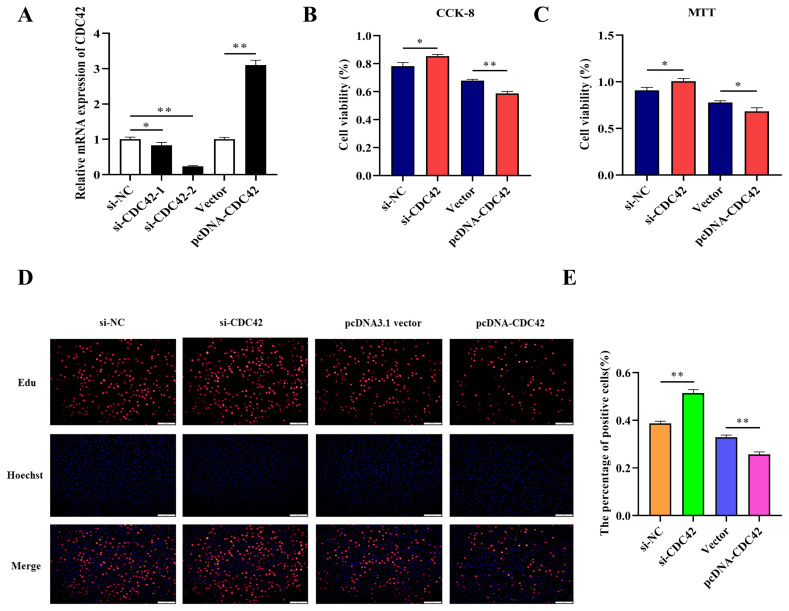
The effect of CDC42 on IPEC-J2 cells proliferation. (A) The interference and overexpression efficiency of *CDC42* gene. (B) The results of CCK-8 assay. (C) The results of MTT assay. (D) The results of EdU assay. (E) The count of EdU positive cells. CDC42, cell division cycle 42; CCK-8, cell counting kit-8; MTT, methylthiazolyldiphenyl-tetrazolium bromide. All ** means p<0.01, * means p<0.05.

To confirm whether miR-185 directly promotes proliferation of IPEC-J2 cells by targeting CDC42, we compared knockdown and overexpression of CDC42 in IPEC-J2 cells. After transfected with si-CDC42-1 and si-CDC42-2, the expression of CDC42 was downregulated by 0.828 and 0.238 fold. So, the si-CDC42-2 was used in subsequent experiments. However, after transfected with pcDNA-CDC42 plasmid, the expression of CDC42 was significantly upregulated (Figure 6A). The CCK-8, MTT, and EdU assays were used to detect cell vitality and cell proliferation, these results showed that knockdown CDC42 promoted cell proliferation, while overexpression CDC42 inhibited proliferation of IPEC-J2 cells (Figure 6B, 6C, 6D, and 6E). Therefore, we hypothesized that miR-185 might directly target CDC42 to promote IPEC-J2 cell proliferation.

## DISCUSSION

Diarrhea is a common disease in pig industry, especially harmful to piglets. Our previous study found that ssc-miR-185 was up-regulated in the resistance group of diarrhea piglets [14]. We speculated that it may play an important role in resisting diarrhea, but the specific target gene is unknown. It is well known that bioinformatics prediction combined with experimental validation is an effective method for screening miRNA target genes. In this study, three softwares: TargetScan, miRDB, and miRanda were used to predict the target genes for miR-185, which could effectively reduce the false positive rate. By finding the intersection, CDC42 was selected as a candidate target gene.

Previous research has shown that CDC42 is a potential target of miR-185. For example, Zhang et al [21] confirmed that CDC42 is a direct target of miR-185 in human hepatocellular carcinoma using luciferase reporter assays. Liu et al [28] showed that miR-185 expression significantly suppressed the RhoA and CDC42 3′UTR activities using a luciferase-reporter assay, and could inhibit the proliferation and invasion of human colorectal cancer cells. Notably, the miR-185 and *CDC42* gene sequence are highly conserved between pig and human. Hence, we assumed that ssc-miR-185 could be binding to the conserved sites of CDC42. In our present study, we found that CDC42-3′UTR contained miR-185 binding site according to the bioinformatics software. The luciferase activity is remarkably suppressed in pmirGLO-CDC42-WT group after transfection with miR-185 mimics. These results indicated that CDC42 was a target gene of porcine miR-185. As a chemokine that mediates tumors, CDC42 can participate in the migration and invasion of various cancer cells [33]. Previous research reported that microRNA-384 inhibits proliferation, migration and invasion of glioma by targeting at CDC42 [34]. Yang et al [35] confirmed that downregulation of miR-25 markedly inhibited A549 cell proliferation, induced G1 cell cycle arrest, by targeting CDC42. In addition, miR-330 regulates the proliferation of colorectal cancer cells by targeting CDC42 [36].

More and more studies have confirmed that miRNA can negatively regulate the expression of target genes. Niu et al [37] demonstrated that ROCK2 was negatively associated with miR-185-5p and promoted hepatocellular carcinoma cell migration and invasion. Fang et al [38] revealed that the expression level of miR-185 and STIM1 were negatively correlated as detected by qRT-PCR and Western blot assays. In this study, the mRNA and protein expression level of CDC42 were dramatically decreased after overexpression of miR-185, which further confirms the targeting relationship between the porcine miR-185 and CDC42. Functionally, miR-185 has been reported to inhibit the proliferation of cancer cells and promote apoptosis. For example, upregulation of miR-185 promotes apoptosis of the human gastric cancer cell line MGC803 [39]. Zou et al [40] found that RKIP through up-regulation of miR-185 suppresses the proliferation and metastasis of breast cancer cell lines. Furthermore, miR-185 can inhibit virus infection through the regulation of immunometabolic pathways [41]. In our present research, we detected the exact function of miR-185 for proliferation and proved that miR-185 promoted the proliferation of normal IPEC-J2 cells. However, whether miR-185 can resist piglet diarrhea caused by pathogenic bacteria infection and inhibit intestinal cell apoptosis requires further research. In summary, our results may provide new insights into the screening of miR-185/CDC42 molecular markers.

## CONCLUSION

In conclusion, luciferase activity, qPCR and Western blot assays displayed that porcine miR-185 can directly target *CDC42* gene. In addition, overexpression miR-185 and knockdown CDC42 can promote cell proliferation of IPEC-J2. However, the detailed regulatory mechanism of miR-185/CDC42 axis in piglets’ resistance to diarrhea requires further investigation.

## Figures and Tables

**Table 1:** Primers information in this study

Name	Accession no.	Primer sequence (5′–3′)	Size (bp)	Region
CDC42-PCR (WT)	NM_001078680.1	F: CCG**CTCGAG**TGTTGCACTGACTGGCTCTG	188	1,373–1,392
		R: ACGC**GTCGAC**CGGGCAGTGGCATCCTTATT		1,560–1,540
CDC42-qPCR	NM_001078680.1	F: GACAGATTACGACCGCTGAGT	151	193–213
		R: TCCCAACGAGCAAGAAAGGAG		343–323
β-actin-qPCR	XM_003124280.5	F: ATATTGCTGCGCTCGTGGT	148	142–160
		R: TAGGAGTCCTTCTGGCCCAT		289–270

The bold and underline indicate restriction enzyme sites.

CDC42, cell division cycle 42; PCR, polymerase chain reaction; WT, wild type; qPCR, quantitative PCR.

**Table 2:** The information of interference RNA sequence

Name	Sense (5′-3′)	Antisense (5′-3′)
si-CDC42-1	CCUACACGACAAACAAAUUTT	AAUUUGUUUGUCGUGUAGGTT
si-CDC42-2	GCUCGUUGGGACCCAAAUUTT	AAUUUGGGUCCCAACGAGCTT
si-NC	UUCUCCGAACGUGUCACGUTT	ACGUGACACGUUCGGAGAATT

CDC42, cell division cycle 42.
